# Ramadan fasting and weight change trajectories: Time-varying association of weight during and after Ramadan in low-income and refugee populations

**DOI:** 10.1371/journal.pgph.0000371

**Published:** 2022-10-26

**Authors:** Daniel E. Zoughbie, Tin Lok James Ng, Jacqueline Y. Thompson, Kathleen T. Watson, Rami Farraj, Eric L. Ding

**Affiliations:** 1 Microclinic International Social Network Research Group, San Francisco, California, United States of America; 2 Institute of International Studies, UC Berkeley, Berkeley, California, United States of America; 3 New England Institute of Complex Systems, Cambridge, Massachusetts, United States of America; 4 Trinity College Dublin, Dublin, Ireland; 5 Institute of Applied Health Research, College of Medical and Dental Sciences, University of Birmingham, Birmingham, United Kingdom; 6 Stanford University, Palo Alto, California, United States of America; 7 Jordanian Royal Health Awareness Society, Amman, Jordan; 8 Harvard School of Public Health, Boston, Massachusetts, United States of America; 9 Federation of American Scientists, Washington, DC, United States of America; University of Bristol, UNITED KINGDOM

## Abstract

Obesity is a significant driver of the global burden of non-communicable diseases. Fasting is one approach that has been shown to improve health outcomes. However, the effects of Ramadan fasting differ in that the type, frequency, quantity, and time of food consumption vary. This phenomenon requires in-depth evaluation considering that 90% of Muslims (~2 billion people) fast during Ramadan. To address this issue, we evaluated the pattern of weight change during and following Ramadan for a total of 52 weeks. The study was conducted in Amman, Jordan. Between 2012 and 2015, 913 participants were recruited as part of a trial investigating the efficacy of a weight loss intervention among those with or at risk for diabetes. Weight was measured weekly starting at the beginning of Ramadan, and changes were analyzed using discrete and spline models adjusted for age, sex, and trial group. Results show slight weight gain within the first two weeks and weight loss in the subsequent weeks. During the first week of Ramadan, the estimate for a weight reduction was 0·427 kg, (95% CI: -0·007, 0·861), increasing to 1·567 kg, (95% CI: 2·547, 3·527) at week 26. There was clear evidence of gradual weight gain from about 4 to 15 weeks and a drop towards the end of the investigation at week 28 (-0·12kg, 95% CI: -0·89, 0·56). Our results show that weight changes occurred during and after Ramadan. Weight fluctuations may affect health risks, and thus, findings from this study can inform interventions. Public health agencies could leverage this period of dietary change to sustain some of the benefits of fasting. The authors (DEZ, EFD) acknowledge the Mulago Foundation, the Horace W. Goldsmith Foundation, Robert Wood Johnson Foundation, and the World Diabetes Foundation. TRIAL REGISTRATION. Clinicaltrials.gov registry identifier: NCT01596244.

## Introduction

As of 2017, approximately 1·8 billion people worldwide observed the Muslim faith [1], and 90% fast during Ramadan [2, 3]. The practise of abstaining from food, water, and other activities is a pillar of the Islamic religious praxis with enormous public health implications that are not yet well understood [4, 5]. Therefore, this period of religious observance represents a large-scale dietary and lifestyle intervention that affects approximately 20% of the world’s population each year [3, 4].

Fasting and intermittent fasting, which generally does not include abstention from water, have been shown to improve health outcomes in chronic diseases [6–8]. By contrast, the long-term health benefits or risks of Ramadan fasting are unclear [5, 9–14]. In observant populations, general practices of Ramadan may include an *iftar* meal to break one’s fast after the sun sets and *suhoor*, a light pre-dawn meal [15]. Ramadan meals may be calorie-dense, water abstention in warm climates may result in dehydration, overeating may occur before bed, and regular sleep patterns may be disturbed [16]. Therefore, the physiological changes associated with Ramadan fasting differ from general, intermittent fasting [17]; the former may have unidentified effects when body functions are disrupted [5, 10].

One driver of the uncertainty concerning Ramadan fasting is its dynamic nature. Since the Ramadan fast lasts from dawn until dusk, fasting from food and water varies significantly depending on geographical location and daylight hours. Unlike fixed-date holidays like Christmas, the Ramadan fasting period is based on the lunar calendar and may occur during the Fall, Winter, Spring, or Summer seasons [2]. Socio-economic status and culture also affect fasting practices: wealthy populations may have the flexibility to reverse their work schedules, sleeping more during the day and eating late into the night [18], whereas less privileged individuals may experience malnourishment during this period [19].

In addition to the diversity of experiences within the defined month of Ramadan, its long-term effects remain unexplored. The implications of dietary and lifestyle changes that persist after the fasting period are especially salient in places like the Middle East, where the rates of obesity and diabetes are rising [20, 21]. In Jordan, the prevalence of diabetes increased from 14% in 1990 to 16% in 2020 [22] and is expected to rise even further [20, 22, 23]. To address this growing burden of non-communicable diseases, government agencies must understand how best to fine-tune population-level diabetes interventions to account for large numbers of diabetic individuals who fast.

Existing strategies [24] aimed at the Ramadan fasting period, such as structured nutrition for diabetes [25], dietary interventions [26], and micro-clinic social network programs [27–30], have demonstrated promising results. However, these studies have not yet elucidated specific patterns of weight change during and after Ramadan that can be enacted upon by public health institutions. This study aims to address this gap by providing preliminary findings on the association between the practise of Ramadan and patterns of weight change over a longitudinal period.

## Materials and methods

### Data collection

This study is a secondary analysis using information from all participants who participated in a clinical trial between 2012 and 2015. Briefly, 1025 volunteers were screened using the study eligibility criteria. 913 participants were recruited between 2012–2013 from three community health centers in Amman, Jordan, as part of a multicenter, 3-arm randomized controlled trial (NCT01596244). Participants in Arm A received “Full Microclinic program (MCP)” with curriculum-activated social network interactions. Participants in Arm B received “Basic MCP” educational sessions. While those in Arm C, the Control Arm, received standard monitoring and care. The primary study evaluated the effectiveness of a 6-month Microclinic Program (MCP) on diabetes management, behavioral risk factors, weight, and metabolic outcomes. Participants in the trial were followed for two years. Men and women 18 years or older were eligible to participate in the trial if they had diabetes or were at risk of diabetes. The study intervention was administered after nurses or study coordinators received signed consent forms from participants. Weight was measured in kilograms using a hospital and homecare scale(Health Scale SVR 160) following a standardized study protocol administered by nurses and study coordinators. The protocol was approved by Western IRB (USA) and the Jordanian National Center for Diabetes, Endocrinology, and Genetics. Details of the study participants are in Table 1, and further information on the trial procedure is in S1 Text [Zoughbie et al., Under review] [31].

**Table 1:** Baseline participant characteristics.

Characteristic, mean (SD) or %	Full MCP	Basic MCP	Control Group
	(n = 540)	(n = 185)	(n = 188)
Age, years	54 · 2	56 · 6	56 · 2
Women, (%)	66 · 5	67 · 0	65 · 0
Weight, kg	85 · 9	85 · 0	86 · 0
Height, m	160 · 3	159 · 6	160 · 3
BMI, kg/m^2^	33 · 6	33 · 5	33 · 4
Systolic blood pressure, mm Hg	129 · 1	131 · 7	132 · 2
Diastolic blood pressure, mm Hg	81 · 3	81 · 0	81 · 3
Mean arterial pressure, mm Hg	97 · 2	97 · 9	98 · 3
HbA1c (%) (SD)	6 · 91	6 · 90	6 · 91
Fasting plasma glucose*, mg/dL	147 · 8	142 · 8	145 · 7

*baseline fasting glucose average of first and second weeks

### Statistical analysis

Descriptive summaries of participant characteristics and outcome measures are presented using means with standard deviations. We combined data for this secondary data analysis and analyzed participants in all study arms as a single cohort. All statistical analyses were performed using R version 3.6.3 and reported following relevant guidelines [32]. We analyzed the time-varying relationship between Ramadan and weight using two multi-level mixed effect modelling approaches–one discrete and one continuous. For both models, we assumed that the relationship between Ramadan and weight depends on the number of weeks since the start of Ramadan. The discrete version of the multi-level model assumes that Ramadan’s relationship with weight changes stepwise with a jump at the end of each week. In contrast, the continuous version of the multi-level model treats time as a continuous variable and models the relationship between Ramadan fasting with weight using a linear spline. The linear spline model assumes a piecewise linear relationship between two adjacent knots. The resulting spline function is continuous with a change in slope at each knot location which captures the change in weight trajectory. The number of knots and the locations of knots for the spline method were determined based on the results from the discrete model. We further controlled for the following covariates—age, sex and trial arms in the model. Sensitivity analysis was performed to investigate the consistency of the results with respect to small changes in the knot locations.

The discrete multi-level model was further extended to allow the possibility that the Ramadan relationship with weight depends on risk factors. In particular, we incorporated the interaction between sex and the number of weeks since the start of Ramadan in the multi-level model to allow the possibility that the relationship between Ramadan and weight depends on sex. Furthermore, the interaction between diabetes and the number of weeks from the onset of Ramadan was added to the multi-level model, allowing the change in weight to depend on whether a participant has diabetes or is at risk of diabetes. We also controlled for the following covariates—age, sex and trial arms in the model.

## Results

**Fig 1:**
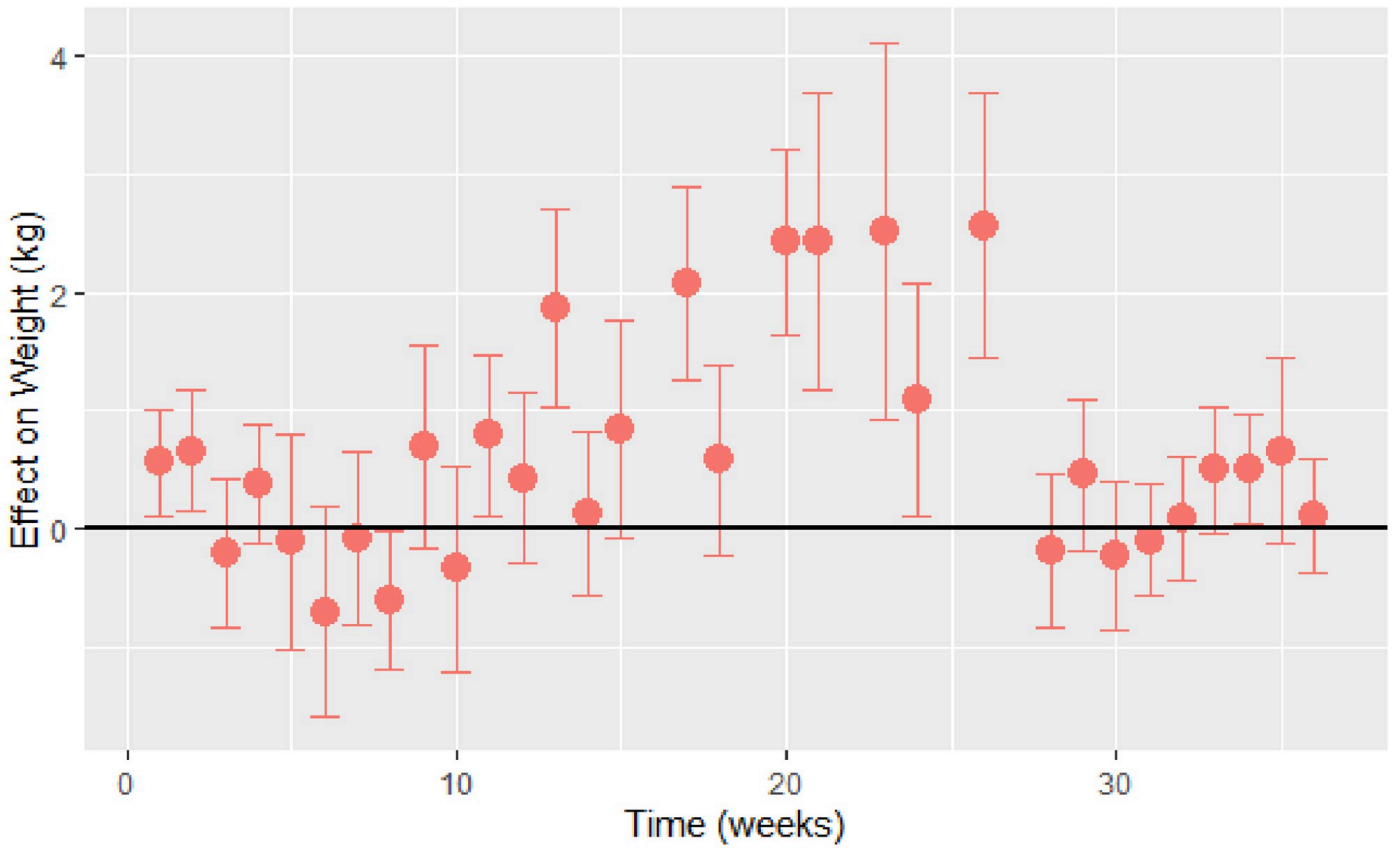
Estimated time-varying effects of Ramadan on weight using the discrete model. Effect on Weight (kg). Time (weeks).

Baseline characteristics of the study participants are presented in Table 1. Further details are in S2 Text. The estimated time-varying effects of Ramadan on weight are shown in Fig 1. We observe a small amount of weight gain in the first two weeks of Ramadan, followed by a gradual decline in weight in the subsequent weeks. By the second week of Ramadan, weight gain was 0·53kg (95% CI: 0·06, 1·01), compared to a weight loss of 0·55kg (95% CI: 0·05, 1·05) by the 8th week, or one month after Ramadan ended. There was gradual weight gain from the 8th week, which continued over the next 18 weeks with an estimated weight gain of 2·54kg (95% CI: 1·57, 3·53) by week 26. A sharp drop of 2·66kg in weight was observed between the 26th (2·54kg, 95% CI: 1·56, 3·53) and the 28th week (-0·12kg, 95% CI: -0·89, 0·56) before it stabilized with average weight returning to similar to baseline values.

**Fig 2:**
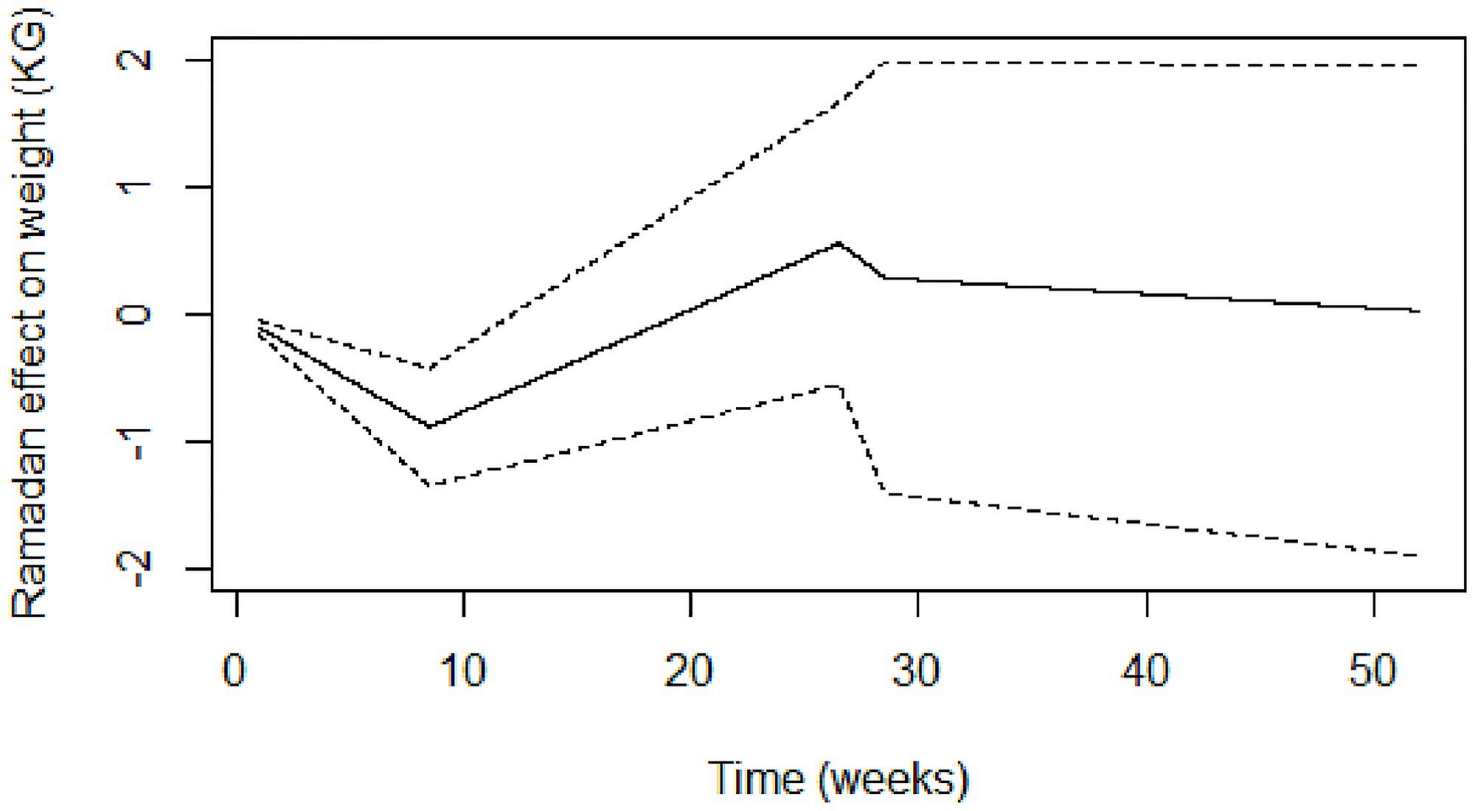
Estimated time-varying effects of Ramadan on weight using the continuous model. Ramadan effect on Weight (kg). Time (weeks).

A similar pattern was observed for the continuous model, as shown in Fig 2. Weight decreased during the first eight weeks of the study (estimated slope, ES: -0·104, 95% CI: -0·158, -0·050) followed by a gradual increase in weight until the 26th week (ES: 0·080, 95% CI: 0·044, 0·116). Another significant drop in weight was observed around week 27 (ES: -0·136, 95% CI: -0·426, 0·154), after which weight changes stabilized (ES: -0·011, 95% CI: -0·021, -0·001).

**Fig 3:**
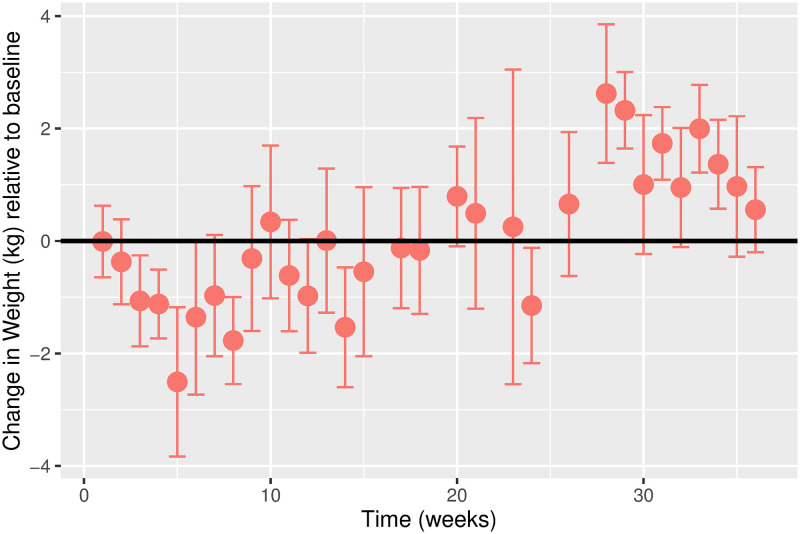
Estimated change in weight (kg) relative to baseline over time in weeks for males. Error bars represent the 95% confidence intervals. Change in Weight (kg) relative to baseline. Time (weeks). Error bars represent the 95% confidence intervals.

**Fig 4:**
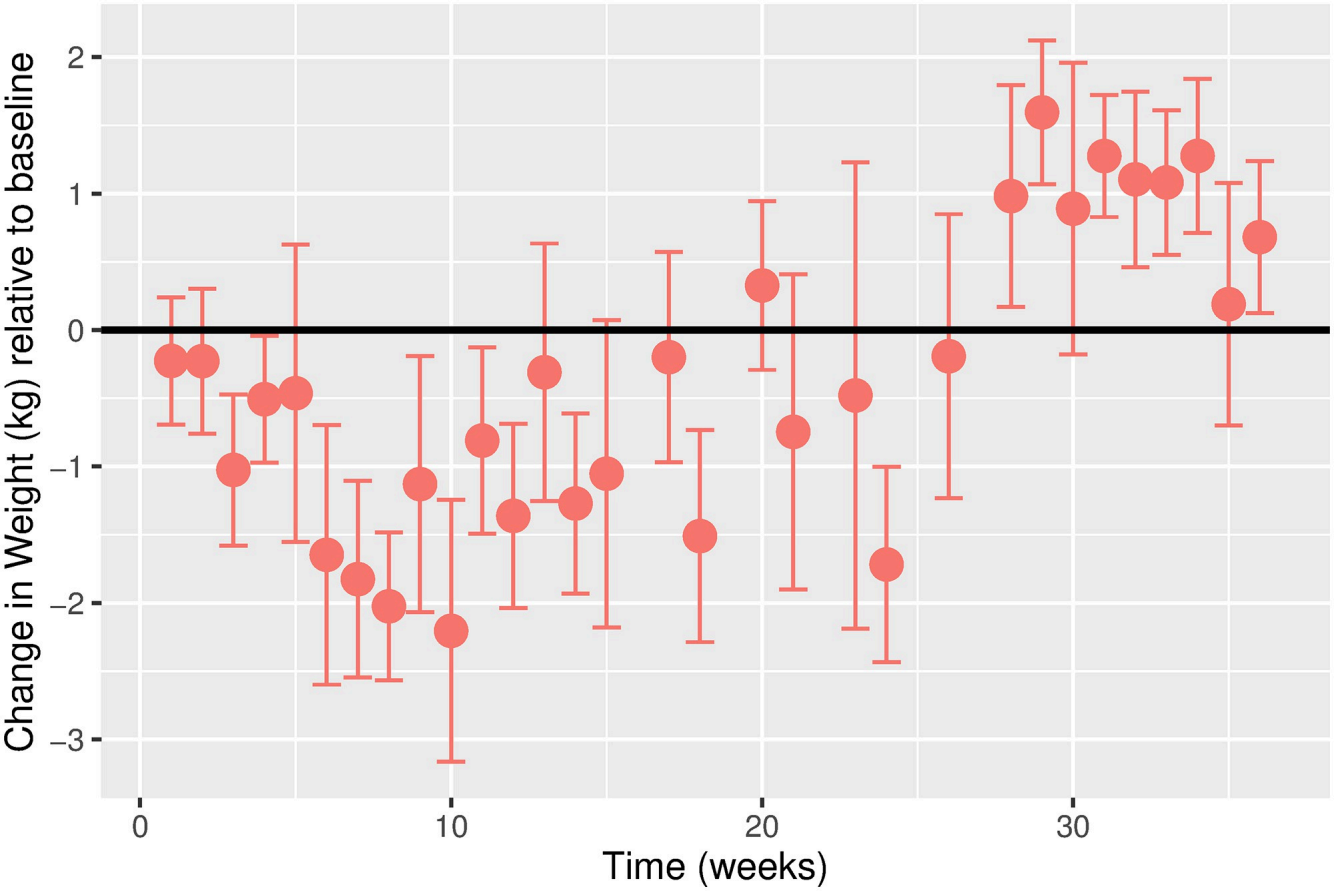
Estimated change in weight (kg) relative to baseline over time in weeks for females. Error bars represent the 95% confidence intervals. Change in Weight (kg) relative to baseline. Time (weeks). Error bars represent the 95% confidence intervals.

The estimated time-varying effects of Ramadan on weight for males and females are shown in Figs 3 and 4, respectively. While the overall weight trajectory is similar for both sexes, there are noticeable differences. We observe a more considerable fluctuation in the weight trajectory for females compared to males. For males, we observe a gradual decline in weight in the first five weeks before a gradual increase. For females, the weight gradually drops in the first ten weeks before an increase is observed. For both sexes, weight fluctuation stabilizes around week 30.

**Fig 5:**
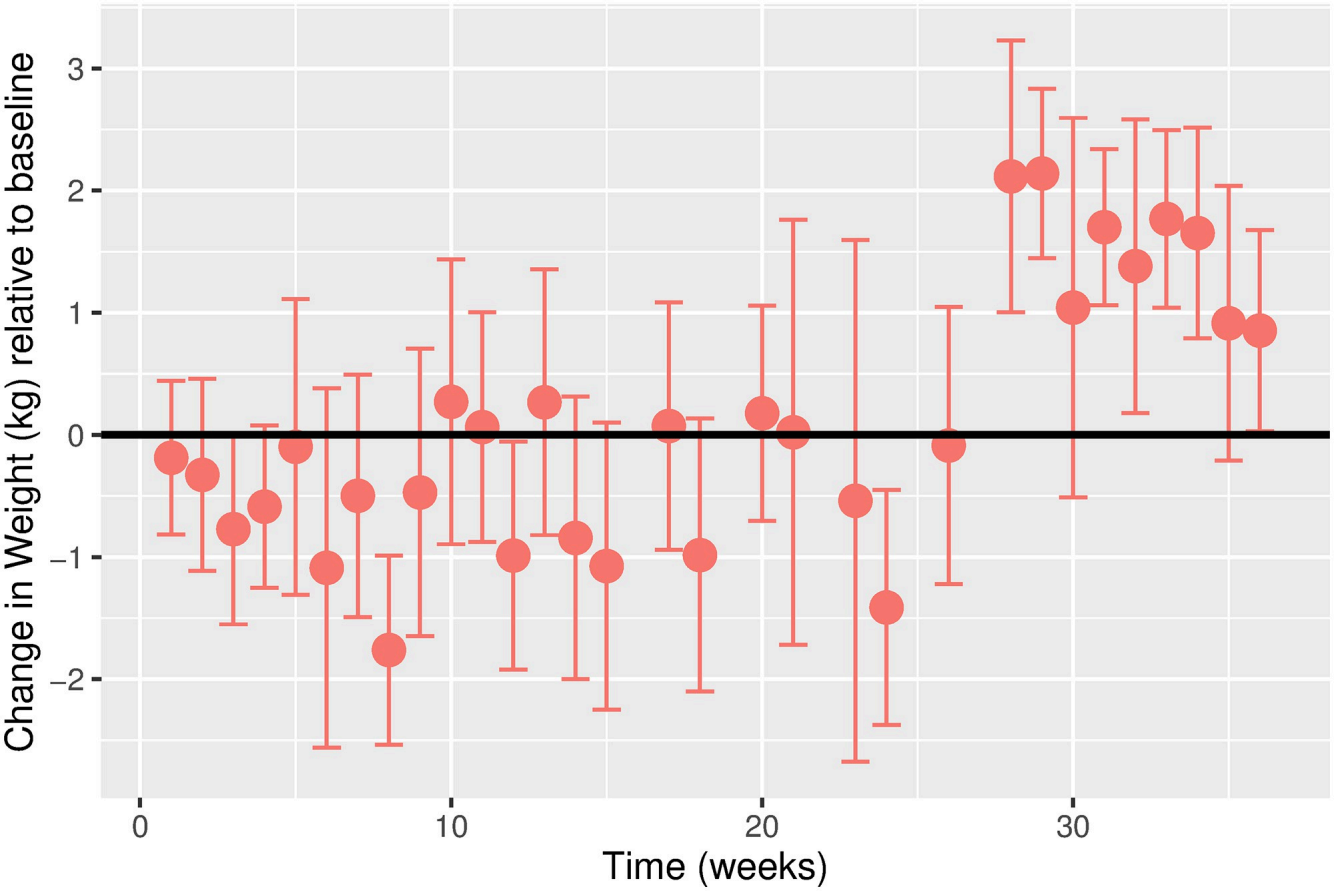
Estimated change in weight (kg) relative to baseline over time in weeks for non-prediabetic and non-diabetic individuals (HbA1c < 5.7%). Error bars represent the 95% confidence intervals. Change in Weight (kg) relative to baseline. Time (weeks). Error bars represent the 95% confidence intervals.

**Fig 6:**
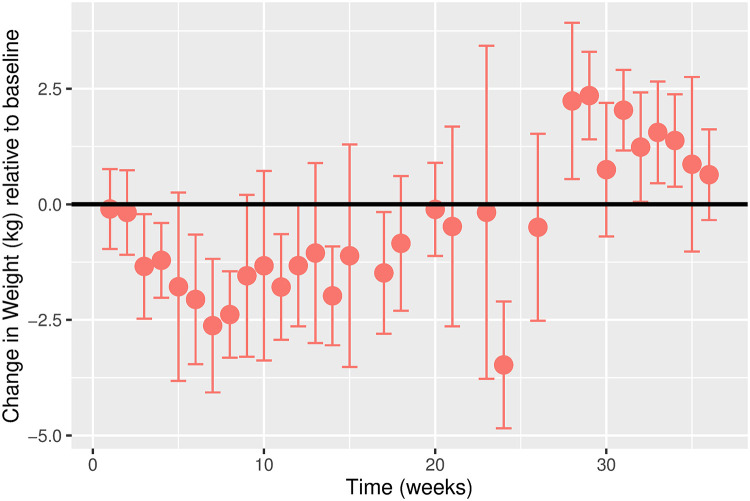
Estimated change in weight (kg) relative to baseline over time in weeks for pre-diabetic individuals (HbA1c between 5.7% and 6.5%). Error bars represent the 95% confidence intervals. Change in Weight (kg) relative to baseline. Time (weeks). Error bars represent the 95% confidence intervals.

**Fig 7:**
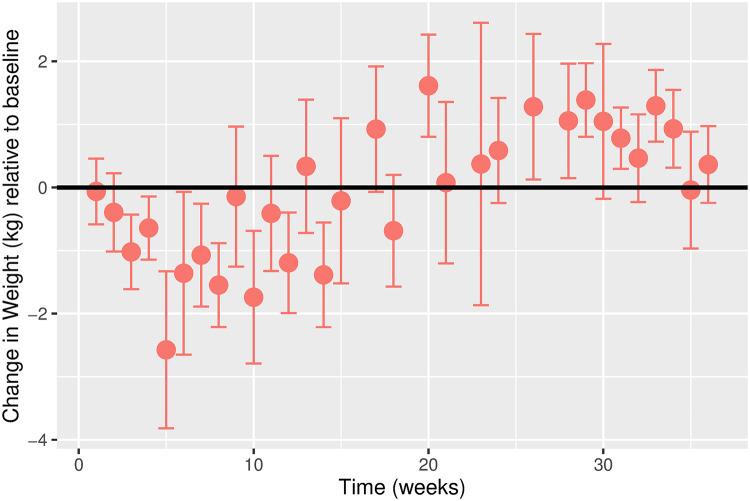
Estimated change in weight (kg) relative to baseline over time in weeks for diabetic individuals (HbA1c > 6.5%). Error bars represent the 95% confidence intervals. Change in Weight (kg) relative to baseline. Time (weeks). Error bars represent the 95% confidence intervals.

The estimated time-varying effects of Ramadan on weight for non-pre-diabetics and non-diabetics (HbA1c < 5·7%), as well as pre-diabetic (HbA1c between 5·7% and 6·5%) and diabetic (HbA1c > 6·5%) individuals are shown in Figs 5–7, respectively. Overall, we observe similar weight trajectories for the three groups of individuals. However, the weight fluctuation for non-prediabetic and non-diabetic individuals is considerably larger than the other two groups. The largest weight gain is observed at week 27 for both non-prediabetic, non-diabetic, and pre-diabetic individuals. In comparison, weight peaked at week 20 for diabetic individuals.

## Discussion

To our knowledge, this is the first study to evaluate the pattern of weight change during and following Ramadan for a total of 52 weeks using data gathered over two years. The study participants consisted of a sample of individuals with diabetes, pre-diabetes, or at-risk adults. Specifically, there was a steady loss in weight until week 8 (0·5 kg), followed by steady weight gain until week 26 (3 kg), then a sharp drop in weight between 26 and 28 (>2·5 kg). From the 28^th^ week onwards, we observe that weight stabilizes. Over time, this fluctuation in weight may be concerning since bodyweight fluctuations have been shown to increase the risk of incident type 2 diabetes, with higher variability increasing risk [33]. The present study can help inform public health organizations of when to intervene and how to tailor interventions [34]. Therefore, an important finding of this study is that it identifies critical turning points associated with fluctuations in weight over a longitudinal period. Our results are similar to previous systemic reviews [11, 13], where Ramadan fasting was shown to reduce a small amount of weight (− 1·022 kg, 95% CI − 1·164 to − 0·880 kg) but only in the short term [35, 36]. However, because of the long-term follow-up in our study, we were also able to show that weight begins to increase shortly after Ramadan ends.

One limitation of our study is that data was collected as part of a clinical trial focused on weight loss, limiting its external validity and comparability with other studies and potentially influencing the weight changes seen. However, despite individuals being enrolled in the trial, a clear pattern of weight change during and following Ramadan was seen regardless of when the participants began the trial, which was on a rolling basis. Also, the study took place in Jordan between 2012 and 2015, with Ramadan overlapping the Summer months of June, July and August. While we would expect similar dynamic patterns to emerge from other Ramadan seasons, our findings are not necessarily generalizable to other times of the year, especially the winter months where days are shortened, and severe weather may alter economic activities. It is also plausible that the patterns of weight change seen in this study were influenced by other factors, such as economic, seasonal, or political factors that change throughout the year.

Another limitation is that as a secondary analysis, we did not explicitly inquire about physical activity levels or fasting adherence during Ramadan among the trial participants. However, the rates of fasting among Muslims is known to be high (90%) [2, 3], and although people with diabetes are generally advised not to fast [37], roughly 80% of people with type 2 diabetes still choose to fast during Ramadan [3]. This, together with reports from the study staff and nurses involved in the participants’ care, means we are confident that the vast majority of participants fasted during Ramadan.

Lastly, this study is not randomized. It is almost impossible to randomize participants into fasting and non-fasting groups because Ramadan is a religious obligation adhered to by most people in Jordan. Consequently, unknown and known confounders such as sex [2], duration of fasts [11], smoking, nutrition, dietary pattern [11], socio-economic status, cultural practices, or customs [38] may confound the association between weight loss and fasting during Ramadan. Furthermore, our findings are not generalizable to Muslims who do not fast during Ramadan and may not directly translate to high-income countries and other geographic regions.

### Policy implications

In a recent priority evaluation for non-communicable diseases [39], obesity was again identified as a significant public health problem that lacks effective population-level interventions. Ramadan presents a valuable opportunity for leaders to develop and implement complementary but effective policies [24]. Our study demonstrates weight fluctuations during and following Ramadan. While previous studies have shown that fasting during Ramadan may improve weight [13], no study has provided longitudinal evidence highlighting key time points during and after Ramadan fasting when the ebb and flow of weight trajectories can be identified. One way to harness the opportunity around Ramadan is to deliver targeted public health interventions, raise awareness within communities [24], and implement Ramadan-focused health monitoring campaigns. Future studies should evaluate the magnitude of the temporal effects of Ramadan on weight and other measures of metabolic health, especially in different geographic, cultural, and seasonal contexts. It would also be helpful for such studies to assess findings across sub-groups of low and high-risk populations to aid in the deployment of population-wide interventions.

## Conclusion

Weight fluctuations were observed during and after the Ramadan fasting period. Public health institutions need to be aware of this pattern to harness possible weight loss effects and protect against weight regain.

## Supporting information

S1 Text Main trial procedure. (DOCX)
