# Correction: Ramadan fasting and weight change trajectories: Time-varying association of weight during and after Ramadan in low-income and refugee populations

**DOI:** 10.1371/journal.pgph.0002126

**Published:** 2023-06-26

**Authors:** Daniel E. Zoughbie, Tin Lok James Ng, Jacqueline Y. Thompson, Kathleen T. Watson, Rami Farraj, Eric L. Ding

There are errors in the article Abstract. The correct Abstract is: Obesity is a significant driver of the global burden of non-communicable diseases. Fasting is one approach that has been shown to improve health outcomes. However, the effects of Ramadan fasting differ in that the type, frequency, quantity, and time of food consumption vary. This phenomenon requires in-depth evaluation considering that 90% of Muslims (~2 billion people) fast during Ramadan. To address this issue, we evaluated the pattern of weight change during and following Ramadan for a total of 52 weeks. The study was conducted in Amman, Jordan. Between 2012 and 2015, 913 participants were recruited as part of a trial investigating the efficacy of a weight loss intervention among those with or at risk for diabetes. Weight was measured weekly starting at the beginning of Ramadan, and changes were analyzed using discrete and spline models adjusted for age, sex, and trial group. Results show slight weight gain within the first two weeks and weight loss in the subsequent weeks. During the first week of Ramadan, the estimate for a weight increase was 0·427 kg, (95% CI: -0·007, 0·861) relative to baseline, compared to an estimated weight reduction of 0·55kg (95% CI: 0·05, 1·05) by the 8th week relative to baseline. There was clear evidence of gradual weight gain from week 8 until week 26 with an estimated weight gain of 2.547 kg (95% CI: 1.567, 3.527) at week 26 relative to baseline. A sharp drop of 2.66kg in weight was observed between the 26th and 28th week before it stabilized. Our results show that weight changes occurred during and after Ramadan. Weight fluctuations may affect health risks, and thus, findings from this study can inform interventions. Public health agencies could leverage this period of dietary change to sustain some of the benefits of fasting. The authors (DEZ, EFD) acknowledge the Mulago Foundation, the Horace W. Goldsmith Foundation, Robert Wood Johnson Foundation, and the World Diabetes Foundation. TRIAL REGISTRATION. Clinicaltrials.gov registry identifier: NCT01596244.
