# Supplementary material for: Ramadan fasting and weight change trajectories: Time-varying association of weight during and after Ramadan in low-income and refugee populations
Source: PLOS Glob Public Health. 2022 Oct 26;2(10):e0000371. doi: 10.1371/journal.pgph.0000371 (PMC10021413; doi:10.1371/journal.pgph.0000371)
Supplement: S1 Text — (DOCX) [file pgph.0000371.s001.docx]

**S1 Text:** Main trial procedure

**Methods**

*Trial Design and Setting*

The trial was a multicenter, three-arm, randomized controlled trial that recruited participants from three neighborhoods in Amman, Jordan. The study protocol was approved by institutional review boards in both the United States (Western Institutional Review Board) and locally (The National Center for Diabetes, Endocrinology, and Genetics, Amman Jordan). All participants provided written informed consent. The trial was rolled out in four waves at three participating centers.

*Participants*

Participants were recruited through the Jordanian Ministry of Health (MoH) care centers using a combination of community outreach campaigns and health center patient recruitment between 2012 and May 2013. Men and women 18 years or older were eligible to participate in the study if they had been previously diagnosed with diabetes, were diagnosed with diabetes or pre-diabetes during recruitment, or were at risk of diabetes. Diabetes and pre-diabetes were confirmed by means of a fasting plasma glucose (FPG) test at recruitment, using criteria of 100-125 mg/dL for pre-diabetes, and 126 mg/dL or higher for diabetes. Risk of diabetes was defined as having a history of diabetes in close family AND being overweight/obese, or as having a family history of diabetes AND having either high BP or high serum cholesterol. Pregnant and/or severely ill participants were not eligible to participate in the study.

*Data Collection*

We collected data on clinical measures (height, weight, waist circumference, blood pressure, HbA1c, and FBG), health knowledge, behavior, and demographic data (including survey questions on diabetes and obesity knowledge, healthcare access, exercise/dietary habits, and education status). The clinical data was collected by trained nurses and trained personnel at the MoH community health centers. The survey and social network data were collected via paper-based surveys, administered by nurses and study coordinators.

*Study Procedures*

Potential participants were alerted to the program either through poster advertisements in neighborhood Ministry of Health clinics, via direct contact by study nurses/clinic staff during a patient clinic visits, or by enrolled MCP participants.^18^ Individuals who indicated interest in participating were invited by nurses to attend a study information session and an eligibility criteria confirmation appointment at one of the study centers. Participants were encouraged to bring family members and close friends who might also be eligible to their appointment. Participants were required to fast before their appointment time. During the appointment, study staff and nurses verified participant eligibility criteria, measured FBG, and administered a short introduction, which described the purpose of the program and aims of the study. After hearing the presentation, all interested individuals who met study eligibility criteria could register. Before registering, any questions about MCP were explained and participants signed a consent form. All participants needed to be able to understand, read, and sign the written consent form.

*Randomization*

We randomized units of family, friends, or single individuals who attended screening together to one of the three trial arms. Each individual who went through randomization was designated a node. Singles in trial Arm A, who were not part of a friend/family unit, were invited, post-randomization, to either invite friends or family (who were subsequently omitted from the analysis) to accompany them to Arm A program activities OR to join another family/friend unit in Arm A. The resulting merged social units were designated “microclinics.” Randomization was stratified by study center (three centers), and study cohort waves (up to four), resulting in a total of nine study center-cohorts.

*Study Intervention*

The trial’s primary interventions were delivered over a timeframe of six months (28 weeks) and involved 14 program sessions (Arms A and B), or 14 concurrent check-in appointments (Arm C), all at parallel time intervals between arms, with the first intervention cohort starting in January 2012. Follow-up post intervention was collected at approximately 2 years, between 21-28 months (median 24 months).

The program’s educational lessons summarised by “4 M’s”: Meals, Movement, Monitoring, Medication, covered a variety of topics, including cooking and physical activity lessons, a field visit to a gym, self-care, and assignments to complete.

Intervention Arm A received the Full MCP educational program. In addition, they worked within small microclinic social groups to complete behavior change assignments inside and outside of the classroom. Before each class began, they were taught a social-network based theory of change as follows: 1) I influence and am influenced by the behaviors of those around me, 2) this can be food or bad, 3) I can improve my own behaviors as well as those in my community.

Intervention Arm B consisted of the Basic MCP intervention in which participants received the same educational instructions as Arm A. Given many people in the program knew each other socially or were related to one another from the wider neighborhood, organic classroom social interactions were inevitable. However, Basic MCP classroom participants were not assigned to structured small group microclinic activities. Classroom discussions were also not initiated by the facilitators, though they were self-initiated by participants.

Hence, the fundamental distinction between intervention Arms A and B consisted in the degree to which group social interaction was embedded in the design of activities and the manner in which this socialization occurred. While both Arms A and B covered the same diabetes education content, notably the four M’s, Arm A was structured to actively promote group-based socialization among friends, family, and classrooms whereas Arm B allowed this socialization to occur more organically.

Arm C, the control group, did not receive any classes; however, participants attended parallel appointments on any weekday, within the same matching week as concurrently run Arm A and B classroom programs, to individually collect their biometric data and lab measurements without any other interactions or programming.

*Sample Size*

Both the detailed curriculum and the treatment schedule are shown in Supplemental Appendix I. Randomization was allocated with the ratio of 3:1:1, with resulting group sizes of n= 540, 185, and 188 in arms A, B, and C, respectively. Sample power calculations for HbA1c and weight change were performed based on sample size n = 500 and estimated effects from pilot Microclinic studies. Conservative power simulations show that our study has over 90% power for less than 2.06% change in weight, and over 80% power for a 0.5% reduction in HbA1c. Additional sample power calculations were performed for HbA1c and weight change by varying the effect magnitudes (Fig 2).

*Biometric and Laboratory Measures*

Data was collected by Ministry of Health nurses and study staff. Height was measured using a hospital and home scale with height meter; weight was measured using a hospital & homecare scale was used to measure height and weight (Health Scale SVR 160). Fasting Plasma glucose was measured via the finger-prick method, using AccuCheck Performa. Digital readings on this device converted blood glucose concentrations to plasma glucose concentrations, conforming to international reporting standards. Omron and A&D blood pressure cuffs were used to measure blood pressure; both types measured systolic blood pressure (SBP) and diastolic blood pressure (DBP) three consecutive times; mean arterial pressure was calculated using the clinical formula MAP = (SBP + (2*DBP))/3. HbA1c levels were tested at the Jordanian MoH Lab Centers via High Performance Liquid Chromatography (HPLC). Laboratory analysis for HbA1c study staff was masked, meaning lab workers were not aware which treatment group the samples came from. No masking took place for other outcome measurements.

*Primary and Secondary Outcomes*

The primary outcomes of interest were the overall changes from baseline across 2 years of follow-up of: 1) body weight; 2) (Fasting Blood Glucose) FBG; 3) HbA1c (glycosylated hemoglobin); and 4) blood pressure, indexed via mean arterial pressure (derived from SBP and DBP). To further understand the potential difference between variations of the Full and Basic MCP versus controls, our secondary aim evaluated the follow-up outcomes of body weight, HbA1c, FBG, and blood pressure between individual trial arms.

*Statistical Analyses*

We structured the trial data as a longitudinal, multi-level, panel dataset. We estimated the differences across trial arms of each endpoint using multi-level longitudinal analysis with hierarchal nesting of individuals (within MCP classrooms, within class days, within community centers, and within temporal cohort waves), and using unstructured covariance for time, and with random intercepts at each hierarchal level. The modelling was performed using the intention-to-treat principle, with relative effects in Full and Basic MCP expressed as difference-in-difference against the controls. The overall net effect across all time periods between the Full MCP and the controls and between the Basic MCP and the controls were analyzed as the joint test of all the time-by-group interaction coefficients. All analyses are adjusted for age, sex, time period, neighborhood, day of the week, and external current event factors (Ramadan and political unrest), and the interactions of the external factors. All statistical analyses were performed using STATA 13.1.
